# Suppression of Modulation Instability Induced Phase Noise in the Long-Haul Phase-Sensitive Optical Time Domain Reflectometry

**DOI:** 10.3390/s22218190

**Published:** 2022-10-26

**Authors:** Yichi Zhang, Qi Zhu, Yang Lu, Zhou Meng, Xiaoyang Hu

**Affiliations:** 1 College of Meteorology and Oceanography, National University of Defense Technology, Changsha 410073, China; 2 Academy of Artillery and Air Defense, Nanjing 210000, China

**Keywords:** phase-sensitive optical time domain reflectometry, distributed acoustic sensing, phase noise, modulation instability

## Abstract

Modulation instability (MI) is the main limitation factor of the maximum optical power in long-haul phase-sensitive optical time domain reflectometry (Φ-OTDR), and induces signal fading and serious phase noise. In this paper, a method of coherent seed injection is proposed to suppress the MI-induced phase noise in long-haul Φ-OTDR. The spontaneous MI is suppressed by stimulating induced MI in an optical fiber. The visibility of the signal in Φ-OTDR is enhanced and the phase noise is suppressed significantly. This paper offers an effective method to increase the maximum input power with the MI-induced phase noise suppressed in the long-haul Φ-OTDR system. As a result, the maximum input power and sensing distance can be potentially increased, which is greatly beneficial to the enhancement of the performance of long-haul Φ-OTDR.

## 1. Introduction

Phase-sensitive optical time domain reflectometry (Φ-OTDR) is a typical distributed fiber optic sensing system that has drawn extensive attention in recent years [1]. Nowadays, Φ-OTDR is widely applied in various fields such as oil exploration, civil structure monitoring, seismic monitoring, acoustic sensing, etc. [2,3,4,5,6]. As the technique has been developed, the extension of the sensing range has become a research hot spot. However, the low reflectivity of Rayleigh scattering makes Φ-OTDR extremely vulnerable to power loss in long-haul optical fibers. The increase in the fiber length leads to serious power loss and signal-to-noise ratio (SNR) decrease, which seriously degrades the performance of the system. To obtain a high SNR and long sensing range, the power loss must be compensated for. Numerous researchers have proposed various schemes to increase the sensing range. By using various amplification techniques and system optimization, the researchers have increased the sensing distance to over 100 km [7,8,9,10,11]. Although the optical amplification techniques can easily increase the input optical power and amplification gain in the fiber, the occurrence of one nonlinear effect, called modulation instability (MI), limits the further increase in the input optical power and amplification gain [12]. Up to now, MI has been the main limitation of the maximum optical power and sensing range in Φ-OTDR and the suppression of it is vital to further increase the sensing range.

Although some MI suppression methods have been proposed in other optical fiber sensing systems, such as narrowband filtering [13], time-and-frequency multiplexing [14], orthogonal polarization pulse injection [15], there are still few reports about MI suppression in Φ-OTDR. In 2013, Hugo F. Martins et al. studied MI-induced fading in Φ-OTDR [12]. In 2016, Maria R. Fernandez-Ruiz et al. studied the probe pulse shapes robust against MI-induced fading; however, the method does not suppress MI but averages the MI-induced fading along the fiber [16]. Recently, a method based on a dual-wavelength laser was proposed to suppress MI in Φ-OTDR, but the method needed two separated lasers and the phase noise was not considered [17]. Up to now, an effective method to suppress MI in Φ-OTDR is still in demand.

In addition, the Φ-OTDR achieves high-performance sensing by accurate phase demodulation. Therefore, phase noise is a key parameter that determines the minimum detectable signal. The occurrence of MI results in the power transference from the center frequency to the low-coherence amplified spontaneous emission (ASE) sidebands. Consequently, the phase noise of Φ-OTDR increases as MI occurs. From this point of view, MI has a significant negative impact on the phase noise of Φ-OTDR, which leads to the deterioration of the ability to detect weak signals. Despite this, no study on the MI-induced phase noise has been reported, while, to the best of our knowledge, nor has a method has been proposed to suppress the MI-induced phase noise in Φ-OTDR.

In this paper, the effect of MI on the phase noise of the long-haul Φ-OTDR system is studied in detail. The results show that MI leads to signal fading and serious phase noise. An effective method of coherent seed injection is proposed to suppress the MI-induced phase noise. A narrow-linewidth optical pulse is phase modulated and injected into the sensing fiber. Then, the induced MI is excited, while spontaneous MI is suppressed significantly. As a result, the phase noise is suppressed effectively.

## 2. Modulation Instability in Optical Fibers

**Figure 1:**
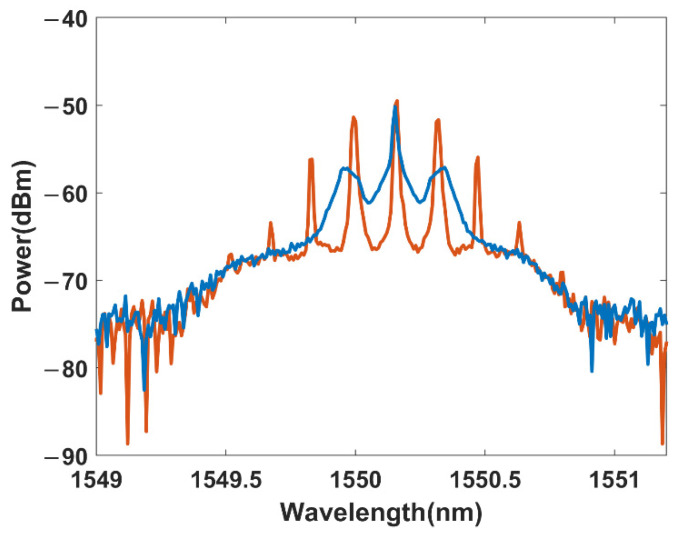
The spectra at the end of 25 km single mode optical fiber when the input power is 400 mW: blue (without seed), red (with seed).

Modulation instability is a nonlinear effect that initiates from the combined interaction of dispersion and nonlinearity in optical fibers. The evolution of MI in optical fibers can be accurately described by the nonlinear Schrödinger equation (NLSE) [18]:

(1) $$\frac{\partial A}{\partial z}+\frac{i\beta_{2}}{2}\frac{\partial^{2}A}{\partial t^{2}}+\frac{\alpha }{2}A=i\gamma {\left|A\right|}^{2}A$$

in which A presents the light field in the optical fiber, $\beta_{2}$ is the anomalous dispersion index, γ is the nonlinear index, α is the linear loss in the optical fiber, z is the distance, and t is the time, respectively. MI leads to the exponential growth of a modulation with frequency $f_{m}$ at the expense of the pump power. Typically, MI initiating from ASE noise is called spontaneous MI, which leads to the generation of symmetric sidebands by amplifying the ASE noise within the MI gain bandwidth $\Omega_{c}=2\sqrt{\gamma P_{0}/\left|\beta_{2}\right|}$. The spontaneous MI sidebands inherit the incoherent nature of the ASE noise, so the coherence of the light is decreased. In optical sensing systems, spontaneous MI leads to the depletion of the signal power and the decrease of the sensing performance. However, the induced MI occurs when some specific seed is added to the pump light. The coherence of the induced MI is related to the seed. Our previous study shows that spontaneous MI can be suppressed effectively by exciting induced MI [19], which leads to significant suppression of the phase noise in discrete fiber sensing systems. The typical spectra at the end of 25 km fiber are shown as Figure 1. When a square pulse with a peak power of 400 mW is injected into a 25 km single mode optical fiber, spontaneous MI amplifies the ASE noise within the MI gain bandwidth and generates spontaneous sidebands. When a 25 GHz phase modulation is added to generate coherent seed around the pump light, the induced MI occurs and generates discrete sidebands. Then, the spontaneous MI sidebands initiating from ASE noise are suppressed significantly. More details can be found in Ref. [19].

## 3. The Modulation Instability-Induced Phase Noise in Long-Haul Φ-OTDR

**Figure 2:**
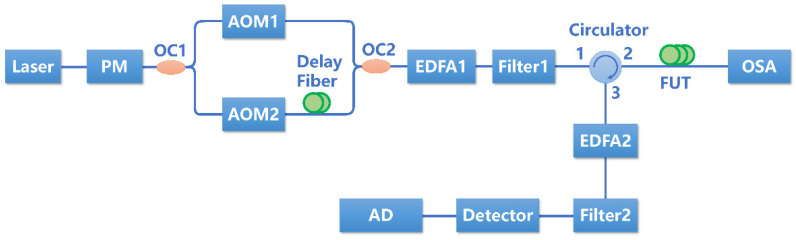
The experimental setup for researching the MI induced phase noise.

The MI-induced phase noise in long-haul Φ-OTDR is studied with the experimental setup shown in Figure 2. A narrow-linewidth laser with a wavelength of 1550.1 nm is used as the light source. The light is modulated by an electronic-optic phase modulator (PM) to generate sidebands around the central frequency. The phase-modulated light is then divided equally by a 50:50 coupler (OC1) and injected into two acoustic-optic modulators (AOM) with frequency shifts of 200 MHz and 220 MHz, respectively. The typical pulse width is set as 200 ns. A 60 m delay fiber is inserted in one path to generate a time delay between two pulses. After combining with another 50:50 coupler (OC2), dual pulses with a time delay of 300 ns and a frequency difference of 20 MHz are generated. It is noted that the dual pulses have the same optical power. Then, the dual-pulses are amplified by an erbium-doped fiber amplifier (EDFA 1) and filtered by a narrow-band filter (Filter 1). After that, the dual pulses are injected into the 25 km fiber under test (FUT) through a circulator. The key parameters of the 25 km FUT are as follows: the nonlinear index γ is $1.3\;W^{-1}{\mathrm{km}}^{-1}$, the linear loss α is −0.19 dB/km, and the dispersion index $\beta_{2}$ is $-21\;{\mathrm{ps}}^{2}/\mathrm{km}$. The optical spectra at the end of the FUT can be recorded by an optical spectra analyzer (OSA). The Rayleigh scattering light is amplified by the other EDFA (EDFA 2) and filtered by a comb filter (Filter 2) to suppress ASE noise. Finally, the filtered light is detected by a photodetector with a bandwidth of 300 MHz and sampled by a high-speed acquisition card. The input power to the FUT can be controlled by changing the gain of EDFA 1. Filter 1 is a narrow-bandwidth filter with a bandwidth of 1 nm. Filter 2 is a multi-channel programmable filter with a minimum bandwidth of 10 GHz.

**Figure 3:**
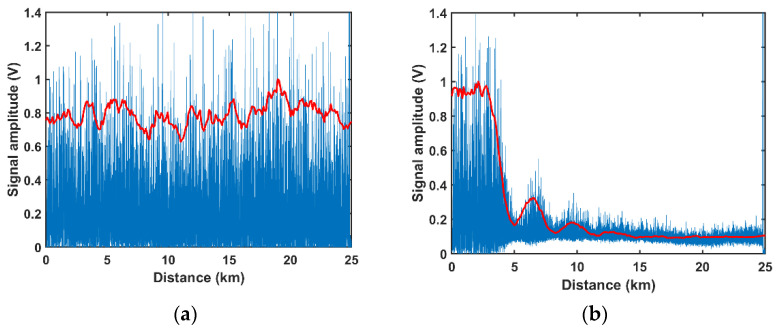
The normalized Rayleigh trace (blue line) and its normalized amplitude (red line) along the FUT when the input power is: (**a**) 150 mW; (**b**) 1240 mW.

When the phase modulation on the PM is turned off, the spontaneous MI occurs in the FUT. The effect of the spontaneous MI in the Φ-OTDR can be studied experimentally. As the Rayleigh scattering signal is filtered with a bandwidth of 10 GHz, most of the spontaneous MI sidebands and other ASE noise can be well mitigated. Consequently, the detected signal is mainly the Rayleigh scattering signal of the central frequency light. The amplitude of the central frequency light can be obtained by measuring the Rayleigh scattering signal [20]. First, we measure the Rayleigh traces with different input powers. To improve the visualization, the linear loss in the optical fiber is compensated for and the amplitudes of the Rayleigh traces are normalized. As shown in Figure 3, the Rayleigh traces show significant difference in various input power situations. When the input power is 150 mW, MI does not occur in the FUT (MI threshold is about 200 mW). As predicted, the Rayleigh traces show random oscillations owing to the spontaneous Rayleigh scattering. The normalized amplitude of the Rayleigh oscillation signal keeps nearly constant along the fiber. When the input power is 1240 mW, the normalized oscillation amplitude of the Rayleigh oscillation decreases with a distance from *z* = 3 km. At about 5 km, the normalized amplitude reaches the minimum value of about 0.16. After 5 km, the normalized amplitude increases reversely. At *z* = 6.46 km, the normalized amplitude attains a maximum value of about 0.32. The normalized amplitude evolution between *z* = 3~6.46 km presents a recurrent period. After *z* = 6.46 km, the normalized amplitude shows similar recurrent evolutions between 6.46~9.64 km and 9.64~12.62 km. After 12.62 km, the recurrent evolution gradually disappears due to the noise-induced thermalization [21]. The points where the normalized amplitudes reach minimum values (5, 8.3, 11.6 km) are defined as the fading points in reference [12]. The visibility and SNR decrease seriously at these fading points.

**Figure 4:**
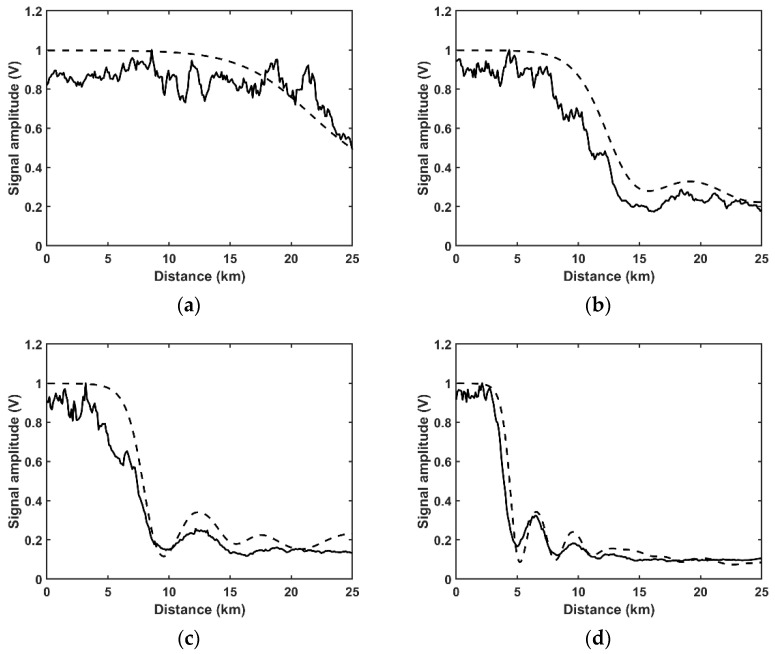
The evolution of normalized amplitude along the FUT in experiment (solid lines) and simulation (dashed lines): (**a**) 374 mW; (**b**) 500 mW; (**c**) 745 mW; (**d**) 1240 mW.

To fully show the effect of MI on the normalized amplitude, the evolutions of the normalized amplitudes along the optical fiber in different input powers situations are shown in Figure 4. The experimental results are shown in solid lines. When the input power is lower than the MI threshold (150 mW), the normalized amplitude keeps nearly constant along the whole 25 km FUT (Figure 3a). When the input power is higher than the MI threshold (374 mW), MI starts to occur and the optical power at the central frequency converts to the spontaneous MI sidebands, which leads to a decrease in the normalized amplitude at the far end of the fiber (Figure 4a). When the input power is 500 mW, the normalized amplitude decreases quickly along the 25 km FUT, and a slight recurrence can be found at the far end of the fiber (Figure 4b). With the further increase in the input power, the recurrent evolution of the normalized amplitude becomes more drastic, and multiple-recurrent evolution can be observed in the FUT. When the input power is 745 mW, two recurrent periods can be found (0~12.6 km and 12.6~18.86 km). When the input power is 1240 mW, at least three recurrent periods can be found (0~6.5 km, 6.5~9.5 km and 9.5~12.9 km). It should be noted that the visibility of three recurrent periods without any distributed amplification reveals the low noise of the experimental system [22]. 

A simulation is conducted to analyze the evolution of the normalized amplitude of the Rayleigh scattering signal by solving the NLSE with the split-step Fourier algorithm. The simulation parameters are set as $\gamma \approx 1.3\;W^{-1}{\mathrm{km}}^{-1}$, α=−1.9 dB/km and $\beta_{2}\approx -21\;{\mathrm{ps}}^{2}/\mathrm{km}$, the power spectrum density of ASE noise is set as −120 dB/Hz. The simulated normalized amplitudes of the Rayleigh scattering signal are shown in Figure 4 with dashed lines. The numerical results agree well with the experimental results. The decrease in the normalized amplitude can be explained as follows: When the input power is lower than the MI threshold, almost all the optical power keeps staying at the central frequency along the whole fiber, so the normalized amplitude is nearly constant in the FUT. When the input power exceeds the MI threshold, MI occurs and the optical power converts from the central frequency to the spontaneous MI sidebands gradually (shown as Figure 4a). With the further increase of the input power, the recurrent evolution of the normalized amplitude can be found. Furthermore, the recurrent evolution becomes more drastic with the increase in the input power and the recurrent fluctuation gradually emerges into noise-induced thermalization. 

Owing to the randomness of the Rayleigh scattering in optical fibers, the phase noise in Φ-OTDR shows a statistical property. The value of the phase noise can be determined by the statistical average result. In this paper, the statistical method in reference [23] is adopted to calculate the phase noise along the 25 km FUT in the system. 

**Figure 5:**
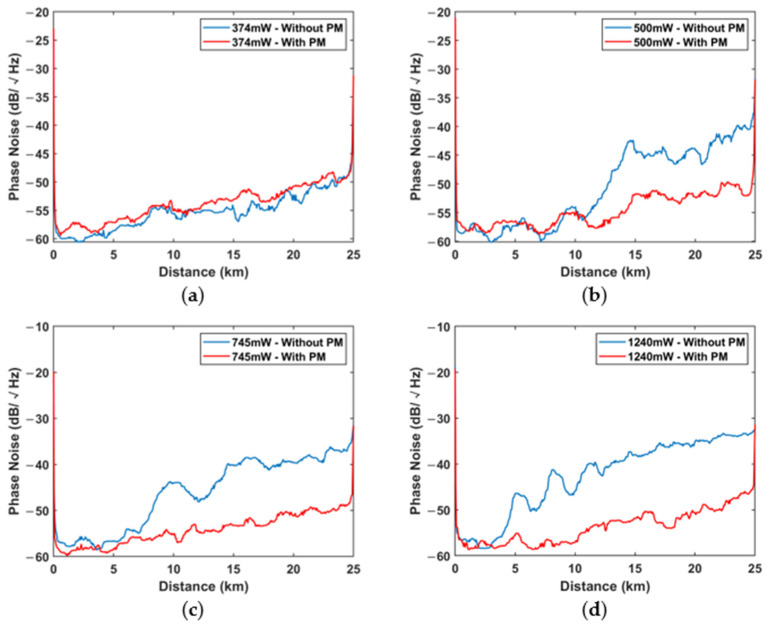
The evolution of phase noise along the FUT in experiment without phase modulation (blue lines) and with phase modulation (red lines): (**a**) 374 mW; (**b**) 500 mW; (**c**) 745 mW; (**d**) 1240 mW.

The evolutions of the phase noise along the FUT in different input powers cases are shown in Figure 5 with blue lines. When the input power is below the MI threshold, the phase noise increases gently along the FUT due to the linear loss of the optical fiber. When the input power is higher than the MI threshold, the phase noise begins to increase steeply at a specific position. With the further increase in the input power, the position where the phase noise starts to increase becomes closer to the near-end. Meanwhile, the evolutions of the phase noise also show oscillation properties in the high input power situations, and the positions of the inflection points of the oscillations correspond to those of the signal fading points shown in Figure 4. The higher the input power, the clearer the oscillation becomes. Three maximum phase noise points are observable at 4.95 km, 8.12 km, and 11.45 km, respectively, when the input power reaches as high as 1240 mW. These positions of the points are almost the same as the signal fading points shown in Figure 3b and Figure 4d, which are located at 4.96 km, 8.157 km, and 11.44 km, respectively. Therefore, it is strongly indicated that the MI-induced signal fading leads to the increase in the phase noise. Most severely, the phase noise at the far end of the FUT reaches as high as −33 dB in the situation where the input power is 1240 mW. 

In conclusion, the occurrence of MI leads to serious phase noise in long-haul Φ-OTDR. Under this condition, the increase in the input power leads to the deterioration of the performance rather than the improvement of the SNR. Therefore, the suppression of the MI-induced phase noise should be considered in long-haul Φ-OTDR systems.

## 4. Suppression of MI-Induced Phase Noise in the Long-Haul Φ-OTDR Systems

The suppression of the spontaneous MI with the coherent seed injection method has been studied in detail in our previous paper. The effect of the method on the spectra and phase noise of the forward transmission light was also shown in reference [19]. However, the effect of the proposed method on the suppression of the phase noise in Φ-OTDR has not yet been revealed. 

### 4.1. The Principle of the Coherent Seed Injection Method

As shown in reference [19], when a narrow-bandwidth light is phase-modulated by the PM, symmetric discrete sidebands are generated around the central-frequency light. As the modulation frequency is near the maximum MI gain frequency, induced MI can be excited significantly. The coherent seeds amplified by the induced MI can be observed from the red line of Figure 1. Compared with the spontaneous MI initiating from the ASE, the induced MI originating from the coherent seed is more competitive. Therefore, the induced MI excited by the coherent seed has more gain, while the spontaneous MI is suppressed effectively. Then, the continuous spectrum (relatively continuous within the gain bandwidth of MI) of the spontaneous MI is replaced by a discrete spectrum composed of the amplified coherent seeds. Finally, the amplified coherent seed can be filtered by Filter 2, which possesses a minimum filtering bandwidth of 10 GHz. In this way, the spontaneous MI as well as the induced phase noise increase can be suppressed.

### 4.2. Phase Noise Suppression with the Coherent Seed Injection Method

Figure 5 shows the experimental results of the phase noise distribution along the optical fiber with/without the coherent seed injection. The blue lines are the phase noise distributions without the coherent seed injection, while the red lines are the results with the coherent seed injection. It is clearly that the coherent seed injection method works very well in suppressing the MI-induced phase noise in Φ-OTDR. In detail, Figure 5a shows the situation where the input power is not so much higher than the MI threshold. In such a situation, MI occurs only at the far end of the FUT (indicated by Figure 4a), and its effect on the phase noise is limited. Therefore, the influence of the coherent seed is also not so significant. As the input power increases, the MI-induced phase noise tends to be prominent. In this case, the suppression effect of the coherent seed on the phase noise is remarkable. It should be noted that the suppressed phase noise distributions are almost the same in different input power cases, which are all close to the linear loss line. The comparison results of the phase noise with and without the coherent seeds, as well as the suppression ratios, are listed in Table 1. The results clearly show that the MI-induced phase noise can be suppressed by the coherent seed injection method. Typically, the suppression ratios of the phase noise at the position of 20 km are about 8.1 dB, 10.8 dB, and 15.5 dB in the situation where the input power is 500 mW, 745 mW, and 1240 mW, respectively.

## 5. Conclusions

In this paper, the effects of MI on the normalized amplitude of Rayleigh scattering light and phase noise in long-haul Φ-OTDR systems are studied numerically and experimentally. The results show that MI induces signal fading and serious phase noise in long-haul Φ-OTDR systems. It is observed that the phase noise distribution has the similar oscillation characteristic to the signal fading, which indicates that the phase noise rise in the high input power situation is related to the MI. A method of coherent seed injection is proposed to suppress the MI-induced phase noise in long-haul Φ-OTDR systems. The experimental results show that the MI-induced phase noise can be suppressed effectively by the proposed method. Typically, the suppression ratios of the phase noise at the position of 20 km are about 8.1 dB, 10.8 dB, and 15.5 dB when the input power is 500 mW, 745 mW, and 1240 mW, respectively. This work provides an effective method to enhance the input power and extend the range of Φ-OTDR.

## Figures and Tables

**Table 1:** The phase noise suppression effects at 20 km position.

Input Power (mW)	Noise without PM (dB)	Noise with PM (dB)	Suppression Effect (dB)
347	−50.8	−50.5	+0.3
500	−44.2	−52.3	−8.1
745	−39.9	−50.7	−10.8
1240	−34.9	−50.4	−15.5

## Data Availability

Not applicable.
